# Integration of Image Pattern Recognition and Photon Sensor for Analyzing Cytokine Gene Expression Using πCode MicroDisc

**DOI:** 10.3390/bios14060306

**Published:** 2024-06-13

**Authors:** On-anong Juntit, Kanokporn Sornsuwan, Umpa Yasamut, Chatchai Tayapiwatana

**Affiliations:** 1 Office of Research Administration, Chiang Mai University, Chiang Mai 50200, Thailand; onanong.j@cmu.ac.th (O.-a.J.); kanokporn_sornsuwan@cmu.ac.th (K.S.); 2 Center of Biomolecular Therapy and Diagnostic, Faculty of Associated Medical Sciences, Chiang Mai University, Chiang Mai 50200, Thailand; 3 Division of Clinical Immunology, Department of Medical Technology, Faculty of Associated Medical Sciences, Chiang Mai University, Chiang Mai 50200, Thailand; 4 Center of Innovative Immunodiagnostic Development, Department of Medical Technology, Faculty of Associated Medical Sciences, Chiang Mai University, Chiang Mai 50200, Thailand

**Keywords:** gene expression analysis, multiplex detection, imaging technology, πCode MicroDiscs assay

## Abstract

Current quantitative gene expression detection in genomic and transcriptomic research heavily relies on quantitative real-time PCR (qPCR). While existing multiplex gene detection techniques offer simultaneous analysis of multiple targets, we present an alternative assay capable of detecting gene expression simultaneously within a single well. This highly sensitive method utilizes πCode MicroDiscs, featuring unique identification patterns and fluorescent detection. Our study compared this multiplex πCode platform with a qPCR platform for profiling cytokine gene expression. The πCode MicroDisc assay successfully demonstrated the expression of polymerization markers for M1- and M2-like macrophages generated from THP-1-derived macrophages in a qualitative assay. Additionally, our findings suggest a pattern agreement between the πCode assay and the qPCR assay, indicating the potential of the πCode technology for comparative gene expression analysis. Regarding the inherent sensitivity and linearity, the developed πCode assay primarily provides qualitative gene expression to discriminate the polarization of macrophages. This remarkable capability presents substantial advantages for researchers, rendering the technology highly suitable for high-throughput applications in clinical diagnosis and disease monitoring.

## 1. Introduction

Gene expression analysis is essential in understanding molecular biology, providing crucial insights into biological processes and diseases. Traditionally, researchers have concentrated on individual genes, forming the bedrock of molecular biology research [1]. Over time, several techniques to detect gene expression levels ranging from singleplex to multiplex technologies have been wildly developed [2,3]. Among the pioneering multiplex techniques, qPCR enables the precise quantification of gene expression levels with exceptional sensitivity and accuracy. Furthermore, continual advancements in probe design and data analysis algorithms have broadened the multiplexing capabilities of qPCR [4,5]. Microarrays, on the other hand, as a high-throughput screening, allow for the analysis of thousands of genes simultaneously. However, this exhibits low sensitivity and specificity when compared to qPCR and requires specialized equipment and expertise [6]. On the other hand, NanoString Technology offers a compelling alternative, combining high sensitivity and specificity with minimal sample volume requirements. This advantage comes at a higher cost than some other techniques [7]. Next-Generation Sequencing (NGS) has revolutionized genomics and transcriptomics, although its high cost and complex data analysis necessitate specialized equipment and expertise [8].

Biomarkers like cytokines are essential for evaluating and monitoring diseases. Macrophages are the main source of cytokines that play pathological roles in various diseases such as infectious diseases, inflammatory bowel disease, rheumatoid arthritis, cancer, and autoimmune diseases [9]. Generally, macrophages can be polarized into two main subtypes: classical (M1) and alternative (M2) macrophages by a distinct stimulus [10]. M1-polarized macrophages are characterized by the production of pro-inflammatory cytokines whereas M2-polarized macrophages produce anti-inflammatory cytokines [11]. Therefore, characterizing macrophage subtypes and their cytokine profiles is crucial for developing cancer therapies and predicting patient outcomes. Quantifying gene expression facilitates the exploration of various human diseases such as inflammation and infection [2,12].

Technologies for cytokine detection and quantification, particularly bead-based methods, are exemplified by the Luminex assay. This assay offers the capability of multiplexing, allowing the simultaneous measurement of up to 100 different cytokines in a single experiment with a small sample size. However, the Luminex platform requires dedicated analysis instruments, which increases the initial costs [13]. Recently, imaging technology offered a novel end-point PCR technique developed by PlexBio (PlexBio Co., Ltd., Taipei, Taiwan). The simplified technique, known as pi-Code (πCode) MicroDisc technology, is based on the Precision Image Code, using an optical imaging fluorescence analyzer [14]. The πCode™ MicroDiscs technology incorporates capture agents, such as nucleic acid probes or antibodies immobilized on its surface, seamlessly integrating into existing molecular or immunoassay protocols. This approach optimizes detection sensitivity and allows for easy adaptation to PlexBio’s automated platform for high-throughput, multiplexed assays. The πCode assay, evaluated against Sanger sequencing, effectively detects KRAS G12/13 mutations and genotypes of hepatitis C virus (HCV), demonstrating high sensitivity and specificity for clinical use [15,16]. Additionally, it detects total HIV-1 DNA and intracellular HIV-1 RNA, holding promise for monitoring HIV-1 reservoir size and activity in cure strategies [14,17]. Notably, the IntelliPlex SARS-CoV-2 Detection Kit, authorized for emergency use by the FDA, qualitatively detects SARS-CoV-2 RNA in respiratory specimens.

In the present study, we aimed to compare the detection methods between the qPCR assay and the πCode end-point PCR. Cytokine gene expression for interleukin 6 (IL-6), interleukin 10 (IL-10), tumor necrosis factor (TNF-α), and glyceraldehyde-3-phosphate dehydrogenase (GAPDH) have been analyzed using singleplex and multiplex assays. This study provides insights into the advantages and limitations of the πCode end-point PCR compared to qPCR for cytokine gene expression analysis, with a focus on its potential applications in research and clinical settings.

## 2. Materials and Methods

### 2.1. Cell Lines and Macrophage Polarization

The THP-1 cell line (Accession Number: TIB-202^TM^) was obtained from the ATCC (Manassas, VA, USA) and was cultured in the Roswell Park Memorial Institute (RPMI) 1640 medium (Gibco, Grand Island, NY, USA), supplemented with 10% heat-inactivated fetal bovine serum (HI-FBS) (Gibco, NY, USA), 100 U/mL of penicillin (Gibco, NY, USA), 100 µg/mL of streptomycin (Gibco, NY, USA), and 2 mM of L-glutamine (Gibco, NY, USA).

For macrophage polarization, 1 × 10^6^ of THP-1 cells were seeded in a 6-well cell culture plate. The cells were polarized into M0 macrophages by incubation with 61.3 ng/mL of phorbol 12-myristate 13-acetate (PMA) (Sigma-Aldrich, St. Louis, MO, USA) for 6 h. The M0 macrophages were polarized in M1-like (M-LPS/IFN-γ) macrophages by incubation with 5 ng/mL of IFN-γ [18] and 10 ng/mL of lipopolysaccharides (Sigma-Aldrich, MO, USA) for 18 h. M2-like (M-IL-4) macrophages were obtained by incubation with 20 ng/mL of interleukin-4 (IL-4) (Invitrogen, Waltham, MA, USA) for 48 h.

### 2.2. RNA Extraction and cDNA Synthesis

Total cellular RNAs were extracted from THP-1-derived macrophages using the RNeasy mini kit (Qiagen, Germantown, MD, USA). Cells were directly lyzed in the well with an RLT buffer, and the RNAs were extracted following the manufacturer’s protocol. RNA quality was assessed by agarose gel electrophoresis. Next, 1 µg of the total RNAs was primed with an oligo(dT)_20_ primer and converted to cDNA using the Superscript^TM^III first strand synthesis system (Invitrogen, MA, USA). The total RNA and primer mixture was incubated with the cDNA Synthesis Mix, followed by the addition of RNase H to remove the RNA.

### 2.3. Gene Expression Analysis Using qPCR

Gene expressions in response to different stimulations in THP-1 cells were quantified using qPCR with specific primers (Table 1) including IL-6, IL-10, and TNF-α. Primer sequences were designed using the online primer design tool offered by GenScript. The recombinant plasmid of IL-6, IL-10, TNF-α, and GAPDH genes (Sino Biological, Beijing, China) were used to generate a standard curve. The copy number of each standard (10^0^–10^6^ copies/µL) was calculated by traditional methods [19] using: number of copies = amount (ng) × 6.022 × 10^23^/length (bp) × 1 × 10^9^ × 660. The synthesized cDNA (50 ng) was used as a template. The amplification step was performed by the SensiFAST™ SYBR^®^ No-ROX Kit (Meridian Bioscience, Cincinnati, OH, USA) using Qiaquant^TM^ 96 (Qiagen, Hilden, Germany). The cycling condition was performed at 95 °C for 1 min, followed by 40 cycles at 95 °C for 15 s and then at 65 °C for 30 s. A melting curve analysis was performed immediately after amplification at a linear temperature transition rate of 0.1 °C/s from 65 to 95 °C with continuous fluorescence acquisition. The copy number of each target gene in the macrophage polarization samples was calculated using the standard curves generated during qPCR. The relative gene expression of each cytokine gene was then normalized to the copy number of GAPDH [20,21] and expressed as the fold change in the gene expression.

### 2.4. Determination of Gene Expression Using πCode MicroDisc Assay

Cytokine gene expression (IL-6, IL-10, and TNF-α) was assessed in THP-1-derived macrophages (M0, M-LPS/IFN-γ, and M-IL-4) using πCode MicroDisc technology. The sets of biotin-labeled-specific primers (Table 1) were used for PCR amplification. Plasmid concentrations of 10^4^–10^6^ copies/µL and cDNA samples were used as a template. PCR was performed using the Taq DNA polymerase kit (Thermo Fisher Scientific, Waltham, MA, USA). PCR amplification was performed under the following cycling conditions: 95 °C for 4 min, followed by 30 cycles of 95 °C for 30 s, 65 °C for 30 s, and 72 °C for 30 s, with a final extension step at 72 °C for 5 min. The 10 µL of biotin-labeled amplicons were observed by agarose gel electrophoresis.

Detection of the cytokine profile was achieved by the hybridization of biotin-labeled amplicons to specific probes coupled to unique πCode MicroDiscs. For the MicroDiscs singleplex assay, the biotin-labeled amplicons were subjected to the hybridization process using 5 µL of THP-1 cDNA for IL-6, TNF-α, and GAPDH or 10 µL of THP-1 cDNA for IL-10. The reaction was performed in a 96-well microtiter plate with 200 MicroDiscs of one πCode probe. The samples were subjected to the IntelliPlex1000 πCode Processor, and the incubation program was 37 °C for 20 min, followed by three washing steps with the washing buffer. The fluorescent reagent of streptavidin-phycoerythrin (SA-PE) was added to the reaction well. Imaging and fluorescence detection of the labeled πCode MicroDiscs were analyzed by the PlexBio 100 Fluorescent Analyzer. The analyzer measured and quantified the mean fluorescence intensity (MFI) corresponding to the IL-6, IL-10, TNF-α, and GAPDH probes. Figure S1 depicts the workflow of the πCode MicroDisc assay.

For the MicroDisc multiplex assay, each PCR product (2.5 μL for IL-6, TNF-α, and GAPDH and 5 μL for IL-10) was pooled before hybridization and performed in the IntelliPlex1000 πCode Processor. A mix of 100 πCode MicroDiscs of the four probes (IL-6, IL-10, TNF-α, and GAPDH) was subjected to a 96-well microtiter plate. The hybridization program was performed as described above. Relative expression levels of mRNA were normalized by GAPDH expression.

## 3. Results

### 3.1. Morphological Changes and RNA Integrity of THP-1 Derived Macrophages

**Figure 1:**
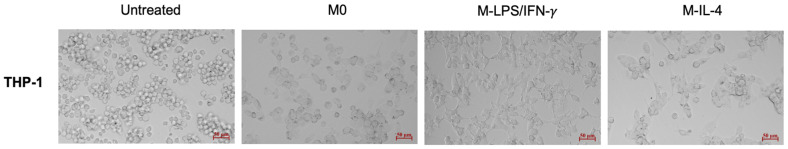
Morphology of THP-1-derived macrophages. After stimulation, cells were observed under inverted microscope. THP-1 cells were incubated with PMA for 6 h to differentiate to M0. Subsequently, M0 macrophages were incubated with LPS and IFN-γ or with IL-4 to polarize to M-LPS/IFN-γ and M-IL-4 macrophages, respectively. Cell imaging was performed using Zeiss Colibri 7 with 20× magnification.

THP-1 cells were polarized into M0, M-LPS/IFN-γ, and M-IL-4 macrophages using different stimuli. After polarization, the morphology of THP-1-derived macrophages was visualized under a microscope (Figure 1). Most M-LPS/IFN-γ and M-IL-4 macrophages had an elongated morphology, whereas almost all of the M0 macrophages showed a round shape. These results suggested the M0 macrophage was polarized to an M1-like macrophage by LPS/IFN-γ and was polarized to an M2-like macrophage by IL-4.

**Figure 2:**
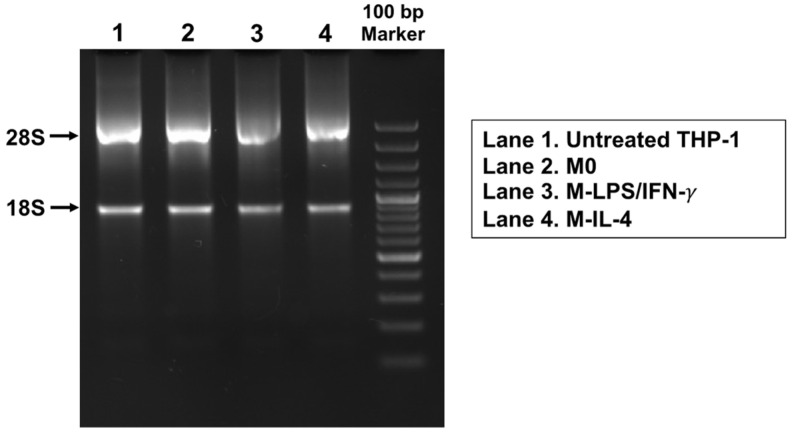
Evaluation of total RNA quality by agarose gel electrophoresis. Total RNA was extracted from untreated THP-1 cells and THP-1-derived macrophages. Distinct undegraded bands around 28S and 18S ribosomal RNA bands were indicated by arrow. The presence of sharp rRNA bands in lanes 1, 2, 3, and 4 with a 28S:18S band ratio of approximately 2:1 demonstrated intact RNA.

Total RNA was extracted from THP-1-derived macrophages, and its quality was determined by agarose gel electrophoresis. The top band represented 28S ribosomal RNA (rRNA) and the bottom band showed 18S rRNA (Figure 2). The presence of two major rRNA bands suggested RNA integrity in all samples.

### 3.2. Cytokine Gene Expression in THP-1 Derived Macrophages Using qPCR

**Figure 3:**
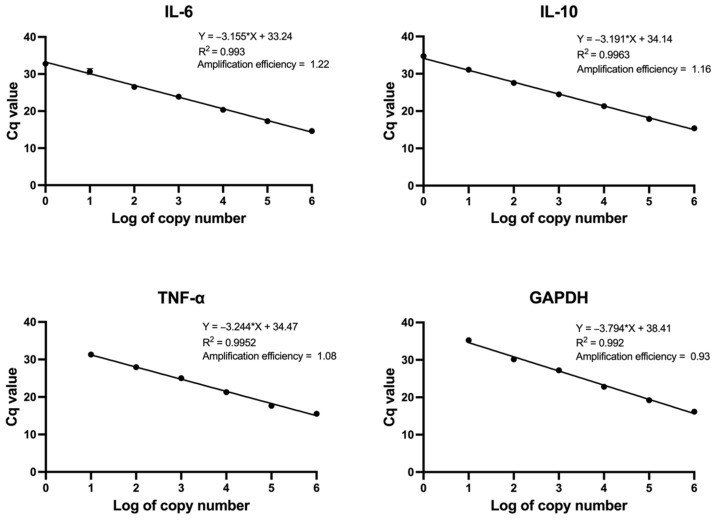
The standard curve of plasmid harboring cytokine genes using qPCR. The plasmid concentrations of 10^0^–10^6^ copies/µL were amplified. Cq values from triplicate measurements were plotted against the logarithm of the copy number of each gene. A linear standard curve was generated with a high correlation coefficient (R^2^ ≥ 0.9).

**Figure 4:**
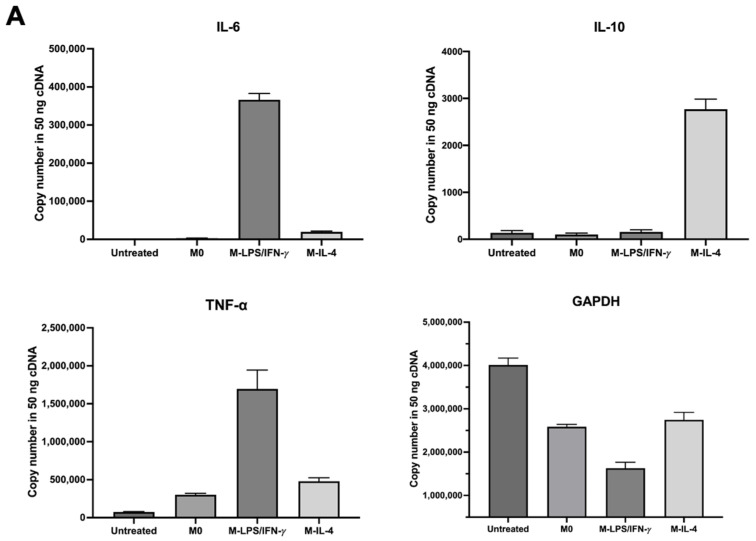

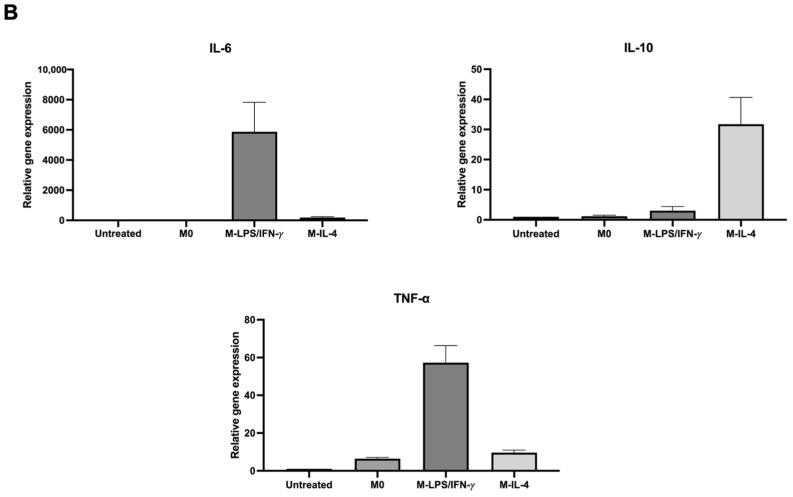
The quantification of cytokine gene expression using qPCR. THP-1 cells were differentiated in M0 and polarized in M-LPS/IFN-γ and M-IL-4 macrophages. Pro- and anti-inflammatory cytokines expressed by THP-1-derived macrophages were determined from a standard curve of qPCR. (**A**) The copy number of each gene was directly calculated from the standard curve and (**B**) the relative gene expression of each cytokine gene was normalized with the copy numbers of GAPDH and calculated as the fold change in gene expression. Each experiment was performed in triplicate and presented as the mean ± SD.

The standard curve of cytokine gene expression was performed using qPCR. The linear range was determined by kinetic amplification of log serial plasmid concentrations from 10^0^ to 10^6^ copies/µL and plotted versus the average Cq values. The results showed that a correlation is greater than 0.95 (Figure 3). The gene expressions of cytokine genes and GAPDH were calculated from the standard curve and demonstrated in Figure 4A. The quantification of IL-6, TNF-α, and IL-10 levels was normalized with GAPDH and was relative to the untreated THP-1, as shown in the fold change of the gene expression (Figure 4B). M1-like and M2-like macrophages were assessed by measuring pro-inflammatory cytokine expressions, including TNF-α and IL-6, and the anti-inflammatory cytokine expression, IL-10, respectively. The results showed that the TNF-α and IL-6 expressions in the M-LPS/IFN-γ macrophages were higher than those in M0. In addition, the expression of IL-10 was increased in the M-IL-4 macrophage. These data suggested that M0 macrophages were polarized to M1-like and M2-like macrophages after stimulation with LPS/IFN-γ and IL-4, respectively.

### 3.3. Cytokine Gene Expression Analysis Using MicroDisc Singleplex Assay

**Figure 5:**
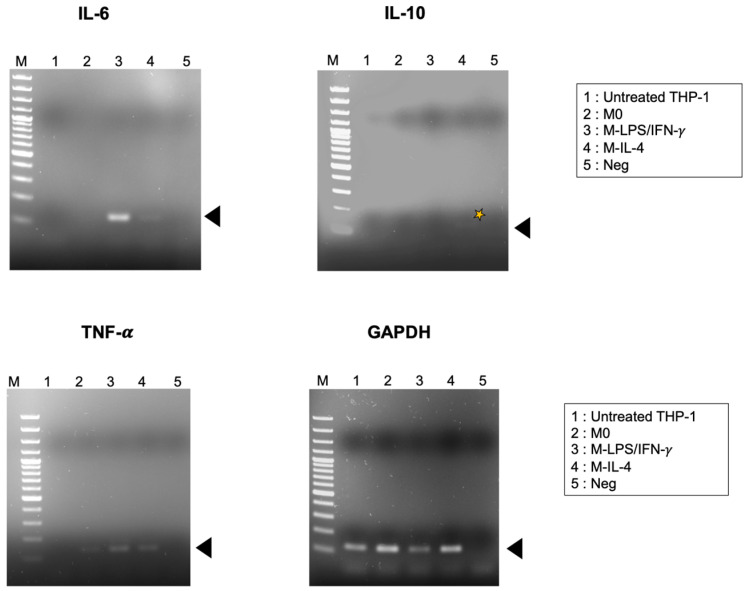
Agarose gel electrophoresis of amplicons for IL-6, IL-10, TNF-α, and GAPDH expressions. To generate M0 macrophage, THP-1 cells were incubated with PMA for 6 h. Subsequently, M0 was treated with LPS and IFN-γ for 18 h or IL-4 for 48 h to establish M-LPS/IFN-γ and M-IL-4 macrophages, respectively. THP-1 and THP-1-derived macrophages were harvested to extract total RNA and synthesized to cDNA. Samples were then subjected to PCR amplification and loaded in agarose gel electrophoresis. Lane 1: PCR product of untreated THP-1 cells; Lane 2: PCR product of M0 macrophage; Lane 3: PCR product of M-LPS/IFN-γ; Lane 4: PCR product of M-IL-4 macrophage; Lane 5: no plasmid control (Neg); M = 100 bp DNA ladder; yellow star = the PCR product of IL-10.

**Figure 6:**
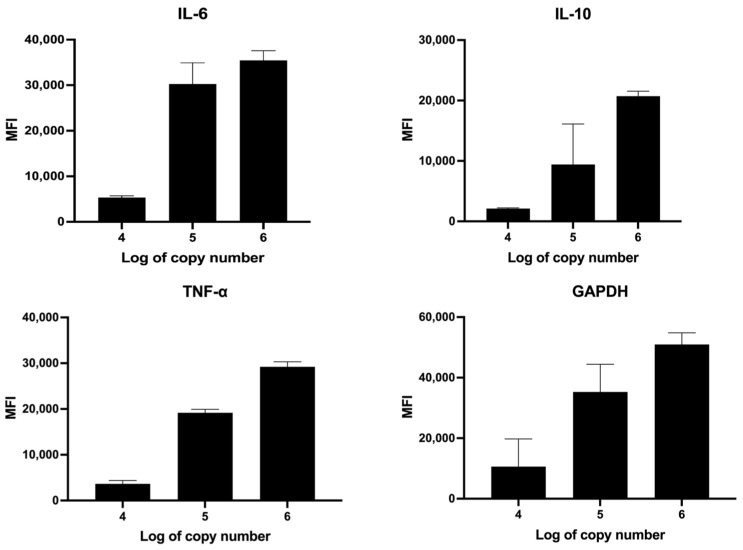
Quantitative detection of recombinant plasmid harboring cytokine genes by MicroDisc singleplex assay. Recombinant plasmid concentrations of 10^4^–10^6^ copies/µL were amplified by PCR. The PCR products of IL-6, IL-10, TNF-α, and GAPDH genes were subjected to hybridization and assessed by the πCode assay. Mean fluorescence intensity (MFI) presented as the mean ± SD from three independent experiments.

**Figure 7:**
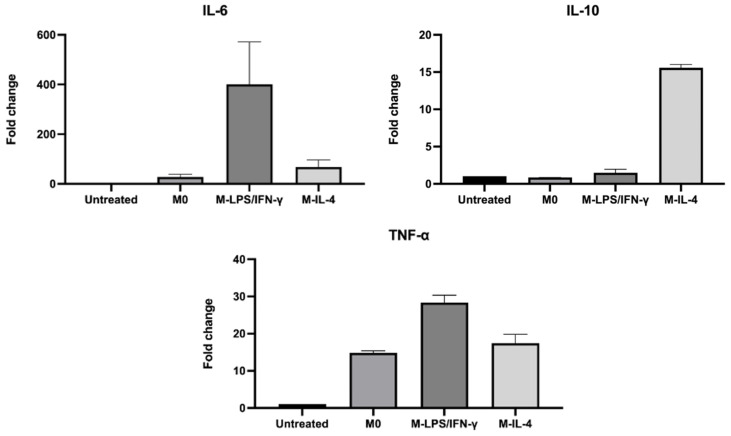
Determination of cytokine gene expression using MicroDisc singleplex assay. Expression of macrophage polarization markers was assessed using single probe labeled with πCode. Mean fluorescence intensity values of IL-6, IL-10, and TNF-α expressions were normalized with GAPDH gene. Expression levels in treated THP-1 were compared with those in untreated THP-1 cells and showed as fold change. Data are stated as the mean ± SD from three independent experiments.

According to the PCR amplification, amplicons were biotinylated. To determine the PCR product band, biotin-labeled amplicons were loaded into the agarose gel electrophoresis and stained with Red Safe (iNtRON Biotechnology, Kirkland, WA, USA). The PCR product sizes of IL-6, IL-10, TNF-α, and GAPDH were approximately 125, 166, 129, and 124 bp, respectively (Figure 5). Subsequently, the PCR product of cytokine genes was subjected to the IntelliPlex^TM^1000 πCode Processor. The mean fluorescence intensity (MFI) of various copy numbers of a recombinant plasmid harboring IL-6, IL-10, TNF-α, and GAPDH was increased in a dose-dependent manner (Figure 6). Furthermore, the cytokine gene expression was evaluated in THP-1-derived macrophages by the MicroDisc singleplex assay. MFI values of IL-6, IL-10, and TNF-α were normalized to GAPDH and were then relative to untreated THP-1 cells to calculate the fold change in expression. The TNF-α gene expression was increased in the M0 macrophage by 15-fold. IL-6 and TNF-α gene expressions were upregulated by 400- and 28-fold, respectively, in M-LPS/IFN-γ compared to untreated THP-1 cells. In M-IL-4 macrophages, the IL-10 gene expression was increased by 15-fold (Figure 7). These findings demonstrated that the MicroDisc singleplex assay can be applied for detecting the cytokine gene expression in THP-1-derived macrophages.

### 3.4. Qualitative Detection of Cytokine Gene Expression Using MicroDisc Multiplex Assay

**Figure 8:**
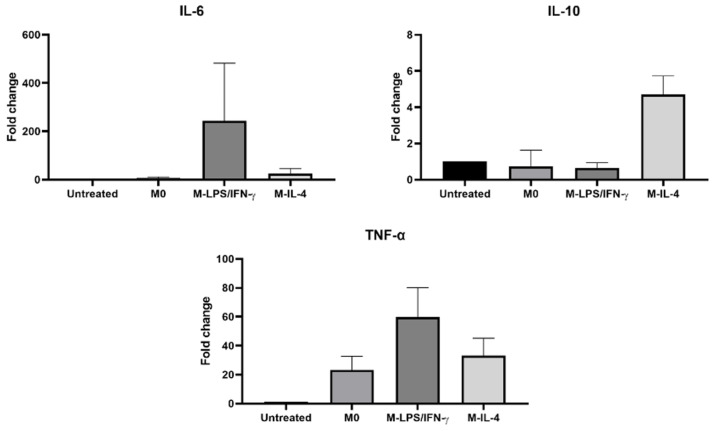
The detection of cytokine gene expression using the MicroDisc multiplex assay. IL-6, IL-10, TNF-α, and GAPDH gene expressions were simultaneously detected using a mixture of four πCodes within a single-well reaction. Mean fluorescence intensity values of the gene expression were normalized by the GAPDH expression. The data were performed in triplicate and presented as the mean ± SD.

To validate multiplex detection using the four probes of πCodes, samples of IL-6, IL-10, TNF-α, and GAPDH were used as templates. Singleplex PCR amplification of four targeted genes was performed in an individual tube. Subsequently, the amplified PCR products are pooled together in a single tube and detected using a mixture of four probes labeled with different πCodes (Figure 8). The MFI value was measured for each πCode after identification recognition. In a study of cytokine gene expression in polarized macrophages, IL-6 and TNF-α expressions were increased by 244- and 60-fold, respectively, in M-LPS/IFN-γ macrophages. IL-10 gene expression was upregulated by 5-fold in M-IL-4 macrophages. These data suggest that the MicroDisc multiplex assay is applicable for detecting cytokine gene expression.

## 4. Discussion

Numerous techniques for gene expression analysis have been employed to assess cytokine gene expression levels. However, a persistent challenge relies on the low sensitivity in the case of a small quantity of samples [22]. While quantitative PCR (qPCR) offers high sensitivity and specificity for mRNA quantification, its multiplexing capability is limited, restricting the simultaneous analysis to 2 to 6 targets [23]. Encouragingly, microbead technology encoded with probes or primers presents a promising avenue for circumventing these constraints of the multiplex qPCR [24]. The QuantiGene™ Plex Assay panel (Invitrogen, MA, USA) is a specific bead-based multiplexed immunoassay system designed for gene expression profiling. This assay utilizes the Luminex platform, enabling the simultaneous analysis of up to 80 RNA targets. Nevertheless, the Luminex platform requires a dedicated instrument equipped with a dual-laser system for proper detection. Therefore, the πCode assay relying on barcoded MicroDiscs was considered for validation in this study. The detection system is composed of a charge-coupled device (CCD) camera for bright field barcode imaging and a single fluorescence signal. The software integrated into the πCode assay allows for the simultaneous analysis of 2 to 200 genes with minimal sample input.

Generally, the M1 macrophage plays an important role in immune function through pro-inflammatory cytokine production. The release of certain cytokines, like TNF-α, IL-1, and IL-6 by these cells, contributes to their microbicidal and tumoricidal functions [25,26]. For M2 macrophages, their functions participate in parasite infection, tissue remodeling, allergic diseases, and angiogenesis [27]. Anti-inflammatory cytokines and chemokines such as IL-10, transforming growth factor beta (TGF-β), and chemokine ligand 1 (CCL1) are upregulated after M2 polarization [28]. In terms of quantifying gene expression with the πCode assay, plasmid concentrations ranging from 10^4^ to 10^6^ copies/μL demonstrated a dose-dependent relationship with MFI values. However, because the assay is end-point-based, the MFI values were insufficient for constructing a reliable standard curve. For the lower than 10^3^ and higher than 10^7^ concentrations, we found that 10^3^ was too low to be reliably distinguished from background noise, likely due to a low signal-to-noise ratio. Conversely, while concentrations higher than 10^6^ did produce a signal, they reached a plateau at 10^7^. This indicates that the assay becomes saturated at higher concentrations. Consequently, qualitative detection is employed instead. In traditional PCR, the intensity of the amplified DNA band visualized on an agarose gel often correlates with the number of starting copies. However, due to limitations in detection sensitivity, bands like the faint IL-10 band in Figure 5 can be challenging to interpret subjectively. Conversely, the πCode assay, employing a high-sensitivity CCD photon sensor, provides a clear and objective fluorescent intensity signal, as demonstrated in Figure 7. This highlights the advantage of the superior sensitivity of the πCode assay. TNF-α gene expression levels were detected in M0 macrophages in both singleplex and multiplex assays. The discrimination of gene expressions following M-LPS/IFN-γ (M1-like) and M-IL-4 (M2-like) macrophage polarization was demonstrated. Increased expression of pro-inflammatory cytokines, IL-6 and TNF-α, suggests that M0 macrophages drive toward the M1-like phenotype as evidenced by real-time PCR and πCode assay results. Furthermore, THP-1-derived macrophages exhibit high IL-10 expression upon IL-4 stimulation indicating polarization toward the M2-like phenotype. While the πCode MicroDisc assay results agree in direction with the qPCR data, directly comparing fold-change values is not possible. This limitation arises from inherent differences in the assays, including sensitivity and the linearity of the πCode method. The detection limit for qPCR is less than 10 copies per reaction, whereas the πCode assay has a detection limit greater than three logs (Table S1). Accordingly, it highlights the reliability of the πCode MicroDisc assay for gene expression analysis.

Although the patterns of the M1-like and M2-like gene expressions were in consensus with qPCR, the fluorescence intensity generated from the multiplex πCode MicroDisc assay was lower than the singleplex experiment (Figure S2). Since the non-biotinylated amplicons were not removed from the PCR products, they may hinder the accessibility of specific biotinylated amplicons to hybridize with the immobilized probes. This phenomenon can impact the actual fluorescence signal intensity. Accordingly, the primers and probes for the πCode assay should be cautiously designed to improve its accuracy. This study primarily focuses on validating the multiplex hybridization reaction using pooled amplicons in the πCode MicroDisc assay. Although it demonstrated promising results, it lacks suitability for application in the diagnostic laboratory. To achieve the linear range that aligns with real-time PCR platforms for the multiplex PCR reaction, a proper PCR cycle needs to be further optimized.

Multiplex real-time PCR assays employ multiple probes, each labeled with a distinct fluorescent dye, which can incur additional costs. Although amplification and probe hybridization are combined in a single step, real-time PCR requires a specialized detector, which may contribute to an increase in the overall system cost. In contrast, the πCode MicroDisc platform necessitates the use of biotinylated primers to obtain PCR products separately from the hybridization step, resulting in a time-consuming process compared to conventional real-time PCR. The πCode assay offers several potential applications for studying multiplexed cytokine analysis, including identifying cytokines stimulated, synthesized, or secreted by immune cells in chronic inflammation and autoimmunity. It allows for the identification of unique cytokine expression profiles associated with different disease states, enabling researchers to analyze upregulated or downregulated cytokines in patient groups. In addition, the assay can be used to monitor cytokine response levels following therapeutic intervention, leading to a more comprehensive assessment of the treatment’s effect. Furthermore, the πCode assay is well suited for analyzing cytokine expression profiles in tumor tissues or immune cell populations isolated from the tumor microenvironment [29]. The current study suggests qualitative detection using πCode MicroDiscs assay relying on the fold change of fluorescence intensity normalized with the housekeeping gene. In the future, suitable standard curves ranging from low to high signal intensity will be established for clinical settings.

## 5. Conclusions

The πCode MicroDisc assay exhibits good concordance with qPCR, indicating its potential as a reliable alternative for qualitative analysis. However, the πCode assay’s limit of detection is demonstrably lower than qPCR, limiting its application in scenarios requiring high sensitivity. Conversely, the πCode assay offers advantages in terms of simplified workflow and the potential for high-throughput multiplexing. These findings suggest that the πCode assay may be a valuable tool for gene expression analysis applications.

## Figures and Tables

**Table 1:** Sequence of primers and probes used in qPCR and πCode assay.

Name	Sequences (5′ to 3′)
IL-6-F	AGACAGCCACTCACCTCTTC
IL-6-R	Biotin tag-AGTGCCTCTTTGCTGCTTTC
Probe_IL-6	AAAACCTCGACGGCATCTCAGCCCT
IL-10-F	CCTGCCTAACATGCTTCGAG
IL-10-R	Biotin tag-GGCAACCCAGGTAACCCTTA
Probe_IL-10	AAAACTCCGAGATGCCTTCAGCAGAGTGA
TNF-α-F	CTGCACTTTGGAGTGATCGG
TNF-α-R	Biotin tag-TACAACATGGGCTACAGGCT
Probe_TNF-α	AAAAAGCCCTCTGGCCCAGGCAGT
GAPDH-F	ACCCAGAAGACTGTGGATGG
GAPDH-R	Biotin tag-TCAGCTCAGGGATGACCTTG
Probe_GAPDH	AAAAGGCGCTGCCAAGGCTGTGGG

## Data Availability

The original contributions presented in this study are included in the article/Supplementary Materials, further inquiries can be directed to the corresponding authors.

## Supplementary Materials

The following supporting information can be downloaded at https://www.mdpi.com/article/10.3390/bios14060306/s1, Figure S1: Schematic of πCode MicroDisc Workflow, Figure S2: Comparison of mean fluorescence intensity values between MicroDisc singleplex and multiplex assays; Table S1: Comparison of fold change in cytokine gene expression between qPCR and πcode MicroDisc assays for macrophage polarization.
