# Supplementary material for: Integration of Image Pattern Recognition and Photon Sensor for Analyzing Cytokine Gene Expression Using πCode MicroDisc
Source: Biosensors (Basel). 2024 Jun 13;14(6):306. doi: 10.3390/bios14060306 (PMC11202078; doi:10.3390/bios14060306)
Supplement: Supplementary file 1 [file biosensors-14-00306-s001.zip › biosensors-3015315-supplementary.pdf]

Integration of Image Pattern Recognition and Photon Sensor for Analyzing Cytokine Gene Expression using πCode MicroDisc

Supplementary Information

On-anong Juntit[1,2,†], Kanokporn Sornsuwan[1,2,†], Umpa Yasamut[2,3,4*], Chatchai Tayapiwatana[2,3,4*]

[1]Office of Research Administration, Chiang Mai University, Chiang Mai, Thailand
[2]Center of Biomolecular Therapy and Diagnostic, Faculty of Associated Medical Sciences, Chiang Mai University, Chiang Mai, Thailand
[3]Division of Clinical Immunology, Department of Medical Technology, Faculty of Associated Medical Sciences, Chiang Mai University, Chiang Mai, Thailand
[4]Center of Innovative Immunodiagnostic Development, Department of Medical Technology, Faculty of Associated Medical Sciences, Chiang Mai University, Chiang Mai, Thailand
*Correspondence: chatchai.t@cmu.ac.th (C.T.); umpa.yas@cmu.ac.th (U.Y.)
†These authors contributed equally to this work.

Supplementary Figure S1: Schematic of πCode MicroDisc Workflow. P.2
Supplementary Figure S2: Comparison of mean fluorescence intensity values between MicroDisc singleplex and multiplex assay. P.3
Supplementary Table S1: Comparison of fold-change in cytokine gene expression between qPCR and πcode MicroDisc assays for macrophage polarization. P.4

Supplementary Figure S1

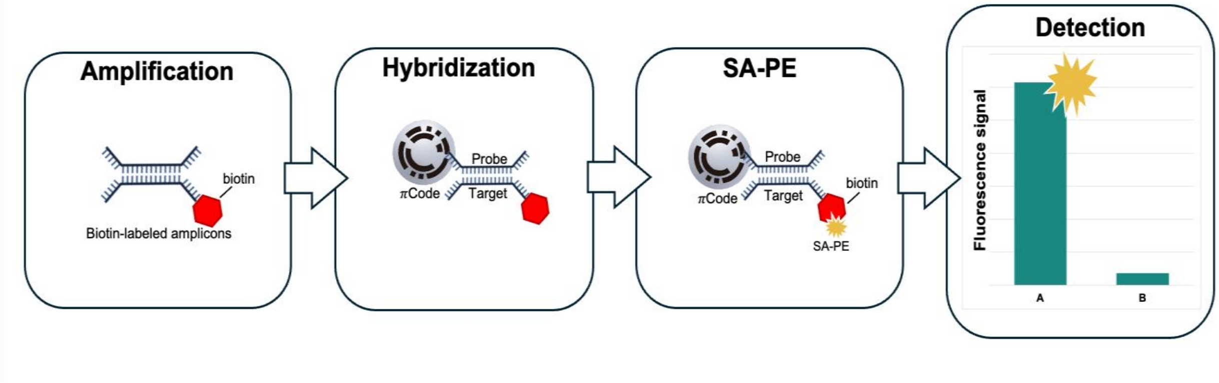

Figure S1. Schematic of πCode MicroDisc Workflow. DNA samples undergo amplification in the initial step using the polymerase chain reaction (PCR). During this process, specific target DNA sequences are selectively amplified, resulting in an increased concentration of amplicons. Biotin-labeled amplicons are generated during this process. The biotinylated amplicons are then hybridized with a specific probe on πCode designed to recognize the target sequence. Streptavidin (SA) conjugated to phycoerythrin (PE) solution is added, leading to SA specifically binding to the biotin-labeled amplicons. The fluorescence emitted by the PE signal intensity is captured in parallel with the MicroDisc pattern recognition.

Supplementary Figure S2

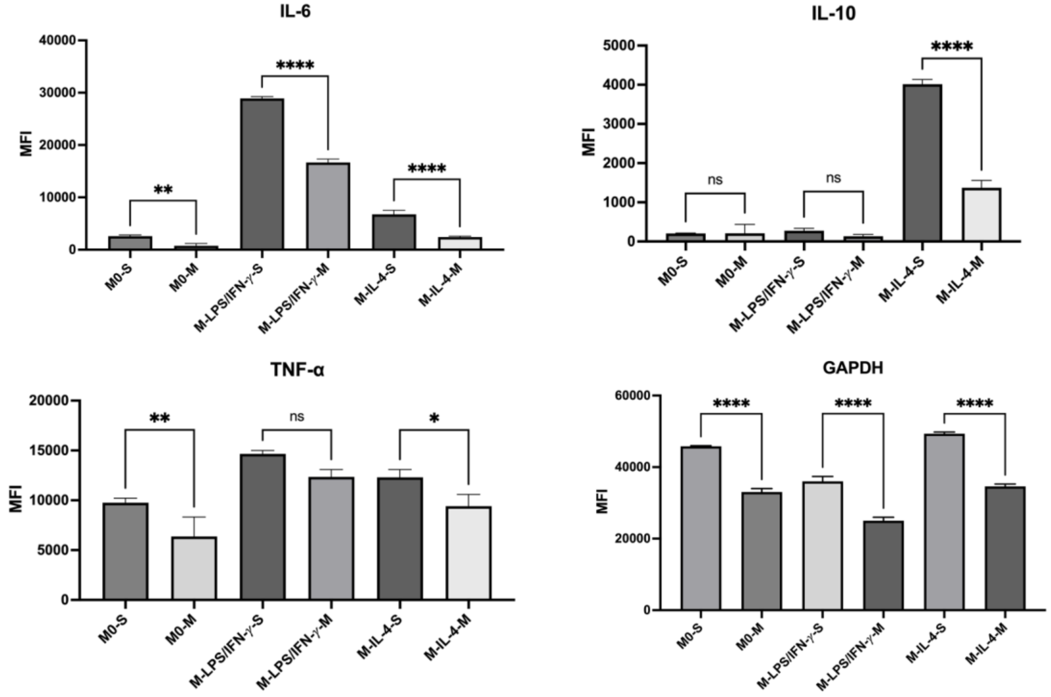

Figure S2. Comparison of mean fluorescence intensity values between MicroDisc singleplex and multiplex assay. Singleplex (S) experiment employed single probe labeled with πCode whereas multiplex (M) used a mixture of four πCodes within single-well reaction. The cytokine gene expression was evaluated. The distinct of fluorescence intensity in THP-1 derived macrophages among M0-S/-M, M-LPS/IFN-γ-S/-M and M-IL-4-S/-M demonstrated in singleplex and multiplex platform. The data are stated as the mean ± SD from three independent experiments. Statistical analysis was performed using one-way ANOVA (ns, p > 0.05; * $p \leq$ 0.05; ** $p \leq$ 0.01; **** $p \leq$ 0.0001) in GraphPad Prism.

Supplementary Table S1

Table S1. Comparison of fold-change in cytokine gene expression between qPCR and πcode MicroDisc assays for macrophage polarization

	IL-6		IL-10		TNF-α	
	qPCR	πCode	qPCR	πCode	qPCR	πCode
**M0**	31.1 ± 5.5	27.9 ± 10.2	1.2 ± 0.3	0.9	6.4 ± 0.7	14.8 ± 0.5
**M-LPS/IFN-γ**	5871.0 ± 1955	400.7 ± 170.7	3.0 ± 1.4	1.5 ± 0.5	57.3 ± 9.1	28.4 ± 2.0
**M-IL-4**	186.1 ± 57.1	68.3 ± 27.7	31.7 ± 8.9	15.6 ± 0.5	9.6 ± 1.3	17.5 ± 2.4
